# Functional reprogramming of peripheral blood monocytes by soluble mediators in patients with pancreatic cancer and intraductal papillary mucinous neoplasms

**DOI:** 10.3389/fimmu.2023.1116034

**Published:** 2023-07-28

**Authors:** Austin M. Eckhoff, Michael C. Brown, Karenia Landa, Ibtehaj Naqvi, Eda K. Holl, David Boczkowski, Ashley Fletcher, Kristen E. Rhodin, Minh Huy Giang, Bruce Sullenger, Georgia M. Beasley, Peter J. Allen, Smita K. Nair

**Affiliations:** ^1^ Department of Surgery, Duke University, Durham, NC, United States; ^2^ Department of Neurosurgery, Duke University, Durham, NC, United States; ^3^ Department of Anesthesiology, Duke University, Durham, NC, United States; ^4^ Department of Pathology, Duke University, Durham, NC, United States

**Keywords:** pancreatic cancer, IPMN, monocyte, innate immunity, TLR

## Abstract

**Background:**

Monocytes and monocyte-derived tumor infiltrating cells have been implicated in the immunosuppression and immune evasion associated with pancreatic adenocarcinoma (PDAC). Yet, precisely how monocytes in the periphery and tumor microenvironment in patients with intraductal papillary mucinous neoplasm (IPMN), a precursor lesion to PDAC, change during disease progression has not been defined. Here we functionally profiled the peripheral immune system and characterized the tumor microenvironment of patients with both IPMN and PDAC. We also tested if sera from patients with IPMN and PDAC functionally reprogram monocytes relative to that of healthy donors.

**Methods:**

Pancreatic tissue and peripheral blood were collected at the time of resection from 16 patients with IPMN and 32 patients with PDAC. Peripheral blood and pancreatic tissue/tumor were immunophenotyped using flow cytometry. Whole blood was plated and incubated with R848 (a TLR 7/8 agonist) or LPS (a TLR4 agonist) for 6 hours and TNF expression in monocytes was measured by flow cytometry to measure monocyte activation. To test if TLR sensitivity is determined by factors in patient sera, we preconditioned healthy donor monocytes in serum from PDAC (n=23), IPMN (n=15), or age-matched healthy donors (n=10) followed by *in vitro* stimulation with R848 or LPS and multiplex cytokine measurements in the supernatant.

**Results:**

TNF expression in R848-stimulated peripheral blood monocytes was higher in patients with low grade *vs* high grade IPMN (65% *vs* 32%, p = 0.03) and stage 1 *vs* stage 2/3 PDAC (58% *vs* 42%, p = 0.03), this was not observed after LPS stimulation. TLR activation correlated with increasing grade of dysplasia from low grade IPMN to high grade IPMN. Serum from patients with IPMN and PDAC recapitulated suppression of TNF induction after R848 stimulation in naïve, healthy donor monocytes.

**Conclusion:**

Peripheral blood monocyte TNF secretion inversely correlates with the degree of dysplasia in IPMN and cancer stage in PDAC, suggesting innate immune reprogramming as IPMNs progress to invasive disease. These effects are, at least in part, mediated by soluble mediators in sera.

## 1 Introduction

Intraductal papillary mucinous neoplasms (IPMN) are cystic precursors to pancreatic adenocarcinoma (PDAC) that account for 15-20% of PDAC cases (1). These precursor lesions are detectable on radiographic imaging and exist on a spectrum of disease from low grade to high grade to those harboring invasive PDAC (2). Early detection and predicting which lesions will develop invasive disease provides an opportunity to intervene prior to the onset of PDAC which is known for its high mortality and immune resistance (1, 3, 4).

Prior work has attributed tumor associated macrophages (TAMs) to PDAC oncogenesis, suggesting that TAMs are critical in creating an immunosuppressive microenvironment and contribute to the extensive desmoplastic remodeling that limits drug delivery (5, 6). However, less is known about the systemic and tumor microenvironment of IPMN. Recently, Bernard et al. performed single cell sequencing of six IPMN tumors and found that as IPMNs progress from low grade dysplasia (LGD) to invasive adenocarcinoma, there is increasing infiltration of myeloid-derived suppressor cells accompanied by T cell depletion (7). Additionally, our own group used spatial transcriptomics to characterize the tumor microenvironment of IPMNs and found that macrophages were generally more abundant in regions of high grade dysplasia (HGD) compared to LGD within the same tumor (8).

Although these prior studies have described the tumor immune cell infiltrate and noted increased myeloid derived cells in HGD IPMN, the functional status of myeloid cells remain undefined, and the systemic immune response is not well described. Elucidating immunologic hallmarks of PDAC development from precursor lesions is expected to inform the development of novel interventions that counter disease progression and/or identification of prognostic biomarkers. Therefore, we sought to comprehensively profile the IPMN tumor microenvironment and systemic immune system with a particular focus on measuring monocyte function. We hypothesized that as IPMNs progress from LGD to HGD, monocytes become more dysfunctional, contributing to innate immune tolerance/reprogramming.

## 2 Materials and methods

### 2.1 Patient recruitment

This study was approved by the Duke University Health System Institutional Review Board (Pro#00100930). Eligible patients were 18 years or older with a diagnosis of IPMN or PDAC who were proceeding to standard of care surgical resection at Duke University Hospital between December 2018 and March 2022. Patients consented to research blood and tumor tissue collection though the Duke BioRepository and Precision Pathology Center (BRPC) at Duke University School of Medicine. Matched blood and tumor tissue from consented patients was obtained by the BRPC and used for examination of the cellular immunome. A board-certified pathologist confirmed tissue diagnosis by separate analysis of tissue sections. Clinicopathological data was prospectively collected by study coordinators and securely stored in a RedCap database.

### 2.2 Blood and tumor processing

Blood was collected on the day of surgery prior to surgical incision. Blood for flow cytometry was obtained by venipuncture and collected in Vacutainer collection tubes containing Heparin or EDTA anticoagulant (BD Biosciences). Blood was rotated on a shaker at room temperature until flow cytometry analysis (10 minutes-4 hours post collection). Blood for serum assays was collected in tubes without anticoagulant (BD Biosciences). Surgically resected tumors were collected and stored in MACS tissue storage solution at 4°C (Miltenyi). Storage time was 1–16 hours post-tumor collection. Tumor tissue was mechanically disrupted with scissors until tumor pieces were 1-2 mm. PDAC was digested using the Tumor Dissociation Kit and Gentle MACS mechanical dissociator (Miltenyi) following manufacturer’s recommendations. IPMN tissue was digested with a separate protocol which is detailed below. Tissue and digestion media (RPMI with 10% FBS, 10mM Hepes, 5mM CaCl_2_ 1x protease inhibitor cocktail, 1x trypsin inhibitor and 100 U/ml DNase) was heated to 37°C for 20-40 minutes until homogenization was achieved. Both PDAC and IPMN tissues were filtered 3 times using a 70 μM cell strainer to remove undigested tissue and dead cell debris and resuspended in 1 ml of PBS. The resulting single cell suspension was immediately analyzed by flow cytometry.

### 2.3 Flow cytometry

All analysis was performed using DuraClone IM (Immune Monitoring) basic antibody panel (CD45, CD3, CD4, CD8, CD19, CD14, CD16, CD56) (9). For peripheral blood analysis, 100 μl of blood was added to the basic panel tube and cells were processed according to the manufacturer’s instructions. In brief, blood was incubated with the antibodies for 15 min in the dark, followed by red blood cell lysis using VersaLyse (Beckman Coulter) for 15 min in the dark. Cells were then washed twice in PBS prior to data acquisition. For tumor cells and tumor-infiltrating immune cells, 1 × 10^6^ cells were added to the basic panel tube and cell staining was performed as described above for peripheral blood. Additionally, propidium iodide (PI, Sigma-Aldrich, St. Louis, MO) was added as a live-dead marker to tumor single cell suspensions. All processed samples were then analyzed on a 13-color CytoFlex flow cytometer (Beckman Coulter). Data was analyzed using Kaluza Software (Beckman Coulter) as previously described (9).

### 2.4 Monocyte stimulation in whole blood

For the monocyte activation assay, fresh whole blood was diluted 1:10 in RPMI and Brefeldin A (BioLegend) was added at 5 µg/ml. Three ml of the blood-RPMI solution was plated in a 12-well plate. LPS, a TLR4 agonist, (100 ng/ml, InvivoGen), R848, a TLR7/8 agonist, (1 µM, InvivoGen) or PBS (negative control) were added to each well and incubated at 37°C for 6 hours. After incubation, cells were harvested from the plate using agitation and cell dissociation buffer.

Cells were analyzed using flow cytometry for surface and intracellular antigens using the Perfix-NC kit (Beckman Coulter). Briefly, 100μl of peripheral blood was incubated with 25 µl of Buffer R1 (fixative agent) for 15 minutes followed by a wash in 2 ml of PBS and centrifugation at 200 ×g for 5 minutes. Cells were then resuspended in 25 μl of 100% fetal calf serum followed by 300 μl of PerFix-NC Buffer R2 (permeabilizing reagent). Next, cells were incubated for 45 minutes with the following antibodies: lineage markers: CD45-KO, HLA-DR-PB, CD14-PC7 and CD16-ECD (Beckman Coulter); activation markers: TNF-APC-AF700 and IL-12-APC. Cells were then washed with 3 ml of Buffer R3 diluted 1:10 in deionized water (wash reagent) and resuspended in 500 µl buffer R3 prior to data acquisition. All processed samples were then analyzed on a 13-color CytoFlex flow cytometer (Beckman Coulter). Data were analyzed using Kaluza Software (Beckman Coulter).

### 2.5 Serum TLR activation assay

HEK-Blue TLR 3, 4, 8, and 9 reporter cell lines were purchased from InvivoGen, and activation in response to control agonists or human sera was determined according to the manufacturer’s instructions. These cells stably co-express a TLR gene and an NF-κB-inducible SEAP (secreted embryonic alkaline phosphatase) reporter gene that can be monitored using SEAP detection media. Briefly, these cells were plated in 96-well plates at a density of 40,000 cells per well and treated for 24 hours with either (1) media alone, (2) a control agonist for each given TLR (Poly I:C [10 µg/ml] for TLR3, LPS [1 μg/ml] for TLR4, R848 [1 µg/ml] for TLR7/TLR8, and CpG ODN [5 μM] for TLR9), (3) cancer patient sera (10 μl), (4) normal human sera (10 μl), (5) media in a final volume of 100 μl. The cell supernatant was subsequently collected and mixed with Quantiblue (InvivoGen) at a 60:40 v:v ratio and incubated for 1 hour at 37°C, after which time absorbance at 650 nm was measured using a Spectramax i3 plate reader (Molecular Devices).

### 2.6 Monocyte stimulation after serum preconditioning

Human peripheral blood mononuclear cells (PBMCs) derived from a LeukoPak (Stemcell Tech) were processed and cryopreserved as previously described (10). PBMCs were thawed and washed in Aim-V media (Gibco), resuspended in Aim-V supplemented with 1 μg/ml DNAse I (Roche) and plated into two 96 well plates (5 × 10^5^ PBMCs per well), incubating at 37°C for 1 hour to allow monocyte attachment. Non-adherent cells were removed and 100 μl of Aim-V media was added per well. Fifty microliters of serum from deidentified patients with PDAC (n=23) or IPMN (n=14), or age-matched healthy donors (n=10) was added per well. Serum was banked under a Duke IRB approved protocol. Monocytes were incubated with sera for 48 hours, followed by removal of sera containing media, gentle washing with 200ul Aim-V media, and replacement of Aim-V media containing mock, R848 (1 μg/ml), or LPS (1 μg/ml). Twenty-four hours later plates were frozen at -80°C. Cytokines were measured in thawed supernatants using the Human Anti-Virus Legendplex (BioLegend). Data were collected on a FACS Canto (Duke Cancer Institute Flow Cytometry Core Facility) and analyzed using the manufacturer’s software. Data were normalized for each cytokine by dividing R848 or LPS MFI values by the respective mock control values to determine fold induction. All experiments/data collection were performed blinded to sera associated disease status.

### 2.7 Statistical analysis

All data were analyzed using GraphPad Prism. Statistical analysis for the flow cytometry data and TLR assays was performed using a Multiple Mann-Whitney test. Tukey’s *post hoc* testing was used to compare cytokine induction after R848 or LPS stimulation between IPMN, PDAC, or healthy donor serum conditioned monocytes. The designated significance level was 0.05 or less.

## 3 Results

### 3.1 Patient characteristics

For flow cytometry analysis, a total of 16 patients between December 2018 and April 2022 were prospectively identified who were undergoing pancreatic resection for IPMN. We obtained IPMN tissue for flow analysis from 14 of those patients and were able to obtain blood for flow cytometry analysis and monocyte activation analysis from 13 patients. Five patients had IPMNs with low grade (LGD) and eleven patients had IPMNs with high grade dysplasia (HGD) or invasive cancer. Basic patient demographics are in **Table 1**.

**Table 1:** Patient demographics and tumor characteristics.

	IPMN Low Grade Dysplasia (n=5)	IPMN High Grade Dysplasia (n=11)		PDAC stage 1 (n=14)	PDAC stage 2/3 (n=18)
Median age: years (IQR)	73 (71-73)	73 (65-80)	Median age: years (IQR)	73 (65-82)	69 (70-74)
Female Sex	3 (60%)	4 (36%)	Female Sex	7 (50%)	7 (39%)
Race			Race		
White	5 (100%)	10 (91%)	White	10 (71%)	14 (78%)
Black	0 (0%)	0 (0%)	Black	3 (21%)	3 (17%)
Other	0 (0%)	1 (9%)	Other	1 (7%)	1 (6%)
Surgery			Surgery		
Whipple	3 (60%)	6 (55%)	Whipple	7 (50%)	14 (78%)
Distal Panc	2 (40%)	5 (45%)	Distal Panc	6 (43%)	4 (22%)
Median Lesion Size, cm	3.8 (2.7-4)	3.6 (3-4.3)	Other	1 (7%)	0 (0%)
Branch Type			Neoadjuvant	11 (79%)	10 (56%)
Main Branch	0 (0%)	5 (45%)	FOLFIRINOX	10 (71%)	7 (39%)
Side Branch	3 (60%)	3 (27%)	Gem/abraxane	3 (21%)	3 (17%)
Mixed	2 (40%)	2 (18%)	Radiation	1 (7%)	0 (0%)
Histology Subset			Stent	6 (43%)	9 (50%)
Intestinal	0 (0%)	3 (27%)	CA 19-9 at Resection (IQR)	22 (13-54)	15 (14-230)
Pancreatobiliary	4 (80%)	4 (36%)	Median Tumor Size, cm (IQR)	2 (1.5-2.8)	3.1 (2.1-3.7)
Gastric	1 (20%)	2 (18%)	Lymph Node Positive	0 (0%)	16 (89%)
Cancer Present within Lesion	0 (0%)	7 (64%)	Lynphovascular Invasion	2 (14%)	13 (72%)
Cancer Stage if Applicable		IA(1), IB (1), IIA(1), IIB (3)	Perineural Invasion	8 (57%)	17 (94%)
Median Follow up, months	32 months	16 months	Median Follow Up, months	4.3	7.7
Cancer Recurrence		3 (27%)	Cancer Recurrence	5 (36%)	6 (33%)
Death	0 (0%)	1 (9%)	Death	3 (21%)	5 (28%)

As expected, a larger percentage of the patients with HGD had main duct (45%) or mixed duct (18%) IPMN than those with LGD. Sixty-four percent of patients with HGD had IPMN lesions that harbored invasive adenocarcinoma. PDAC AJCC staging was as follows: IA (1), IB (1), IIA (1), IIB (2). After a median follow-up of 24 months, three out of five patients with PDAC had recurred, and one of the patients from the HGD cohort had died from recurrent PDAC.

As a positive control, we enrolled 32 patients with a diagnosis of PDAC between December 2018 and April 2022. We divided this cohort by AJCC 8^th^ edition stage at the time of resection (stage 1, n = 14; stage 2/3, n = 18). Basic patient demographics and clinical characteristics are shown in **Table 1**. The majority of patients in both the stage 1 and stage 2/3 cohort had received neoadjuvant chemotherapy (stage 1 - 79% *vs* stage 2/3 - 56%) but only one patient in the stage 1 cohort had received radiation. After a median follow-up of 7 months for the PDAC cohort, 11 (34%) patients had recurrence and 8 (25%) had died from disease.

### 3.2 HGD IPMN is associated with attenuated TNF induction by monocytes after TLR7/8 stimulation

**Figure 1:**
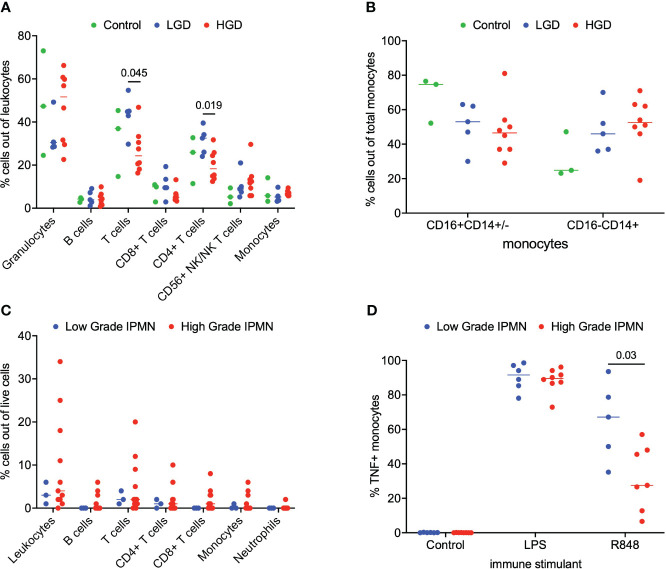
Analysis of immune cell subsets in peripheral blood and IPMN tumor. **(A)** Flow cytometry analysis of immune cells subsets in blood samples from LGD, HGD and healthy volunteer (control). **(B)** Comparison of monocyte subsets (CD16+CD14+/- and CD16-CD14+) in blood from LGD, HGD and healthy volunteer (control). **(C)** Flow cytometry analysis of LGD and HGD IPMN tumor. **(D)** Analysis of monocyte function in whole blood from patients with LGD and HGD post-stimulation with LPS, R848 and negative control (mock). P-value shown obtained with Mann-Whitney test.

We analyzed matched blood and tumor tissue from patients with IPMN of varying degrees of dysplasia. **Figure 1A** depicts results from the whole blood analyses. We examined lymphocytes including B cells (CD19+), T cells (CD3+), CD3+/CD4+ T cells, CD3+/CD8+ T cells, and NK/NKT cells (CD56+). Total monocytes were then separated into classical monocytes (CD16-CD14+) and intermediate/nonclassical monocytes (CD16+CD14+/-), shown in **Figure 1B**. Additionally, because we analyzed whole blood, we were able to examine populations that are excluded when analysis is conducted on peripheral blood mononuclear cells (PBMCs), namely granulocytes (**Figure 1A**). Such phenotyping analyses revealed that patients with LGD had a higher percentage of T cells and CD4+ T cells when compared to patients with HGD in the peripheral blood immune cell populations **Figure 1A**. Otherwise no significant differences were observed in peripheral blood immune cell populations between patients with LGD and HGD. Representative analysis and flow cytometry gating strategy is presented in **Supplementary Figure 1** (**Figure S1**).

Next, we examined the tumor microenvironment in patients with IPMN. Surgically resected fresh tumor tissue (matched to blood from the same patient) was processed into a single cell suspension within 16 hours of tumor harvest followed immediately by flow cytometry. **Figure 1C** shows basic analysis of all tumor-infiltrating lymphocytes within IPMN tissue. Dead cells were excluded and CD45 was used to distinguish immune cells from tumor cells followed by examination of CD4+ T cells, CD8+ T cells, and CD19+ B cells. We collected data from a total of 14 patients presenting with low grade (n=3) and high grade (n=11) dysplasia. Overall leukocyte infiltration in the analyzed tumor accounted for a median of 3% (interquartile range (IQR) 2-9%) of total live cells. T cells accounted for most of the immune infiltrate and accounted for 56% (IQR 43-65%) of the total immune infiltrate. No significant difference between LGD and HGD was observed in total leukocyte infiltrate or any of the immune cell subsets. Representative analysis and flow cytometry gating strategy is presented in **Supplementary Figure 2** (**Figure S2**).

To study the innate immune system and its ability to respond to immune agonists (stressors), we performed a monocyte activation assay wherein peripheral blood was treated with R848, a TLR7/8 agonist, and intracellular TNF production was measured by flow cytometry. We were particularly interested in the monocytes’ response to the TLR7/8 agonist since TLR7/8 has been shown to increase tumor cell proliferation in human PDAC and a TLR7/8 agonist has been shown to prolong survival in a PDAC mouse model (11–13). A statistical difference was found in the monocyte activation between LGD and HGD IPMN patient samples. **Figure 1D** indicates that peripheral blood monocytes from patients with LGD IPMN had greater monocyte activation (LGD 67% *vs* HGD 28%, p = 0.03). These data may imply that peripheral monocytes from patients with HGD IPMN are tolerized to TLR 7/8 activation relative to patients with LGD IPMN. Representative analysis and flow cytometry gating strategy is presented in **Supplementary Figure 3** (**Figure S3**).

### 3.3 Monocytes from patients with later stage PDAC mount weaker TNF responses to TLR7/8 stimulation

**Figure 2:**
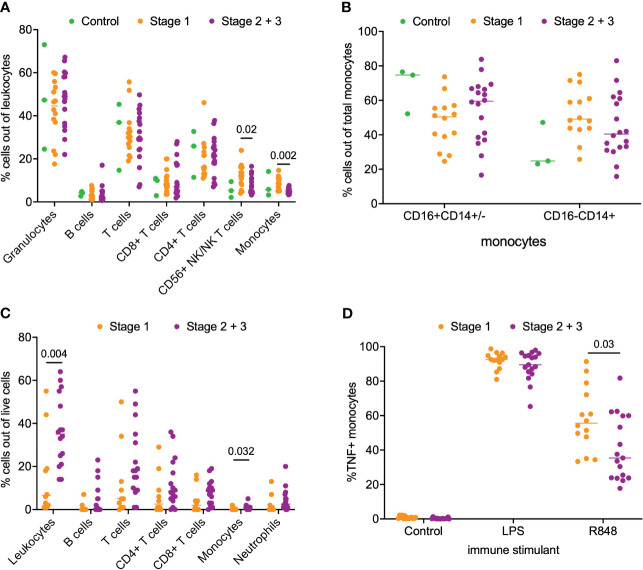
Analysis of immune cell subsets in peripheral blood and PDAC. **(A)** Flow cytometry analysis of immune cells subsets in blood samples from stage 1, stage 2/3 and healthy volunteer (control). **(B)** Comparison of monocyte subsets (CD16+CD14+/- and CD16-CD14+) in blood from stage 1, stage 2/3 and healthy volunteer (control). **(C)** Flow cytometry analysis of stage 1 and stage 2/3 PDAC. **(D)** Analysis of monocyte function in whole blood from patients with stage 1 and stage 2/3 PDAC post-stimulation with LPS, R848 and negative control (mock). P-value shown obtained with Mann-Whitney test.

Next, we asked if a similar phenotype of monocyte activation existed in patients with PDAC. For this analysis, we separated the patients into stage 1 (n=14) and stage 2/3 (n=18) PDAC. We analyzed matched blood and tumor tissue from patients with PDAC. Phenotyping of immune subsets in peripheral blood revealed that CD56+ NK/NKT cells (p = 0.02) and monocytes (p = 0.002) were significantly enriched in the stage 1 patients compared to the stage 2/3 patients. However, no difference was observed in the peripheral monocyte subsets (CD16+CD14+/- and CD16-/CD14+) between stage 1 and stage 2/3 PDAC patients (**Figures 2A, B**). Phenotyping of the PDAC tumor microenvironment showed that leukocytes accounted for a median of 26% (IQR 14-43%) of total live cells within the tumor (**Figure 2C**). Overall leukocyte tumor infiltration was significantly higher in stage 2/3 PDAC compared to stage 1 PDAC (stage 1 6% *vs* stage 2/3 35%, p = 0.004). T cells accounted for most of the immune infiltrate and accounted for 67% (IQR 52-78%) of the total immune cells. Monocytes accounted for very little of the immune cell tumor infiltration and had greater tumor infiltration in stage 2/3 PDAC tumors than in stage 1 PDAC tumors (Stage 1 0.1% *vs* Stage 2/3 0.8%, p = 0.03) (**Figure 2C**).

Next, we examined monocyte responsiveness in the peripheral blood of patients with PDAC using the same TLR agonist that was used for IPMN functional analysis. Notably, as shown in **Figure 2D**, we observed patients with stage 1 PDAC had greater monocyte activation compared to patients with stage 2/3 PDAC (p = 0.03). This result mirrors our IPMN data – patients with higher grade disease, whether IPMN or PDAC, have peripheral blood monocytes that show diminished TNF induction after TLR 7/8 stimulation. Extended data in **Supplementary Figure 5** (**Figure S5**) shows TNF alone, TNF+IL12 and total TNF expression in monocytes treated with agonists for IPMN and PDAC.

### 3.4 TLR Activation is induced by sera from patients with IPMN and PDAC

**Figure 3:**
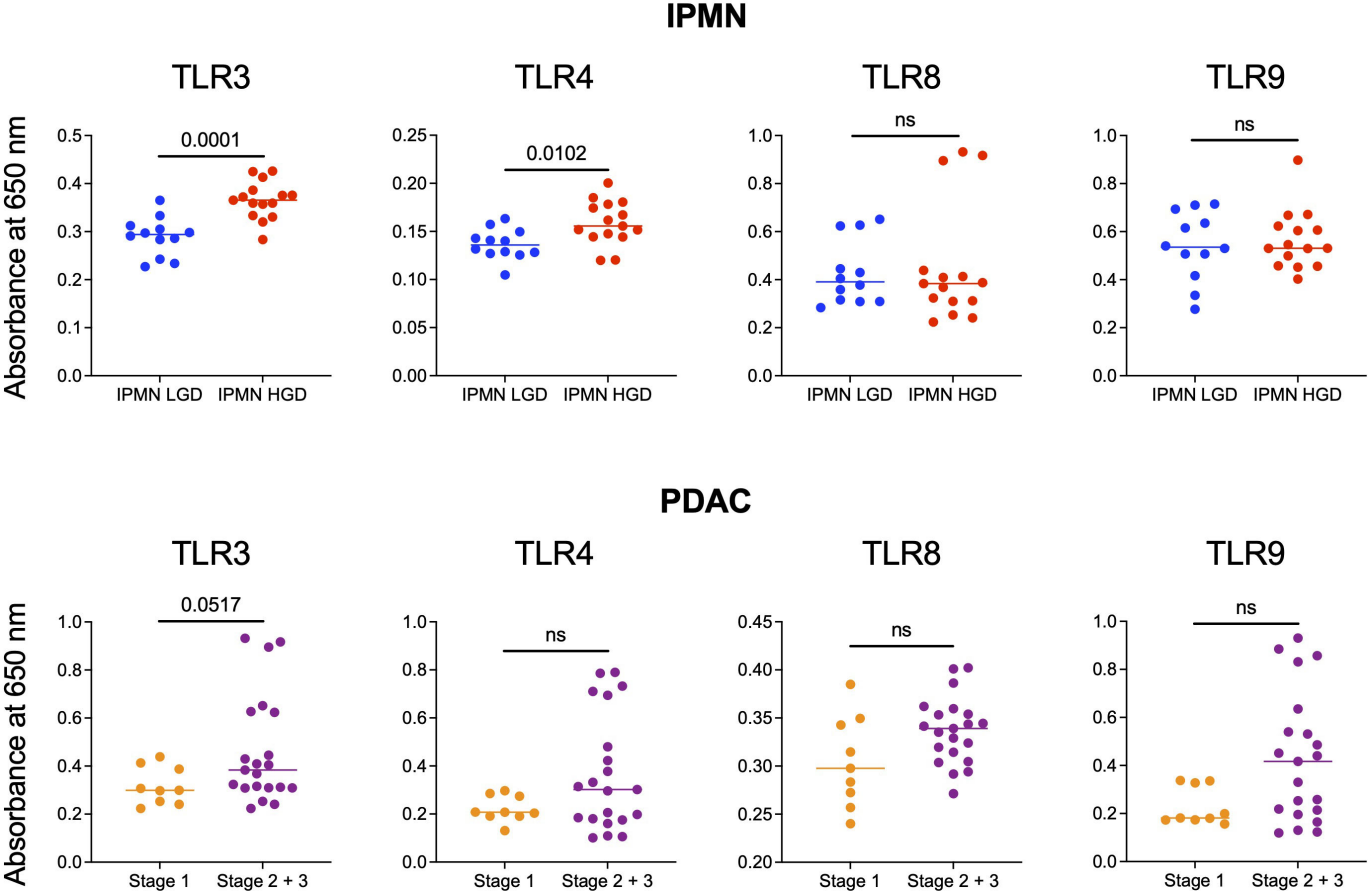
Comparison of the ability of serum from patients with LGD *vs* HGD IPMN and patients with stage 1 and stage 2/3 PDAC to stimulate TLRs 3, 4, 8, and 9. In the IPMN groups, serum from patients with HGD stimulate TLRs 3 and 4 more significantly than patients with LGD. Stimulation of TLRs 8 and 9 are otherwise not significantly different between serum from patients with LGD *vs* HGD. For patients with PDAC, serum from patients with stage 2/3 PDAC trended towards higher TLRs 3, 4, 8, and 9 stimulation when compared to serum from patients with stage 1 PDAC. P-values shown obtained with Mann-Whitney test; ns= not significant (p>0.05).

Monocytes and their subsets are key sensors of and responders to TLR-mediated inflammation and thus status of monocyte function could reflect the degree of systemic TLR-mediated inflammation. In a recent study, we observed that excessive TLR-mediated inflammation affects monocyte function and induces tolerance (14). Thus, we measured the ability of patients’ serum to induce TLR activation in TLR reporter cells to determine if generalized TLR mediated inflammation corresponded with monocyte function and could contribute to peripheral monocytes tolerization in patients with high grade IPMN and later stage PDAC. We hypothesized that the serum of patients with HGD IPMN would elicit greater TLR activation at baseline than those with LGD. Using TLR reporter cell lines, we found HGD samples have higher amounts of TLR activation ability than LGD IPMN for TLR3 (0.365 *vs* 0.294, p = 0.0001) and TLR4 (0.156 *vs* 0.136, p =0.0102) (**Figure 3**). However, we did not find any difference in activation of TLR8 and TLR9 when comparing LGD and HGD IPMN samples.

Additionally, we performed TLR activation assays using the serum from patients with PDAC. We did not find a statistically significant difference in TLR activation between stage 1 PDAC *vs* stage 2/3 PDAC. However, across TLR3 (p = 0.05), TLR4 (p = 0.3), TLR8 (p = 0.8), and TLR9 (p = 0.6) stage 2/3 trended towards greater activation (**Figure 3**). To summarize, we observed higher TLR-mediated inflammation and reduced monocyte function in the blood of patients with HGD *vs* LGD IPMN and a similar trend in the blood of patients with higher *vs* lower stage PDAC. **Supplementary Figure S6** shows extended data with positive and negative controls for IPMN and PDAC analysis.

### 3.5 Sera from patients with IPMN and PDAC reprograms TLR responses in monocytes

**Figure 4:**
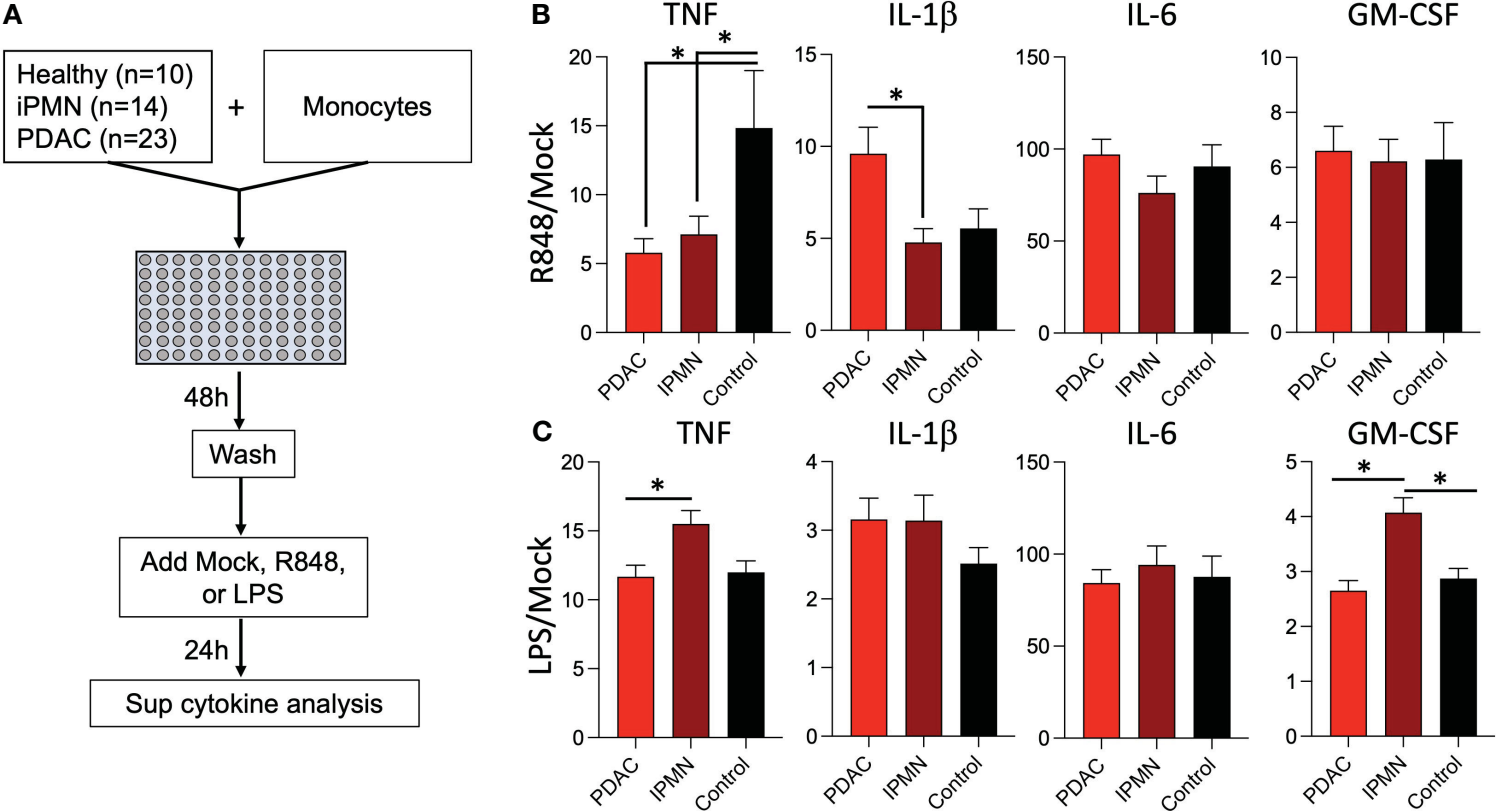
Sera from patients with IPMN and PDAC reprograms TLR responses in monocytes. **(A)** Healthy donor monocytes were co-cultured with 30% serum in media from healthy donors or donors with IPMN or PDAC for 48 hours. The monocytes were washed with media and treated with mock, R848 (1 µg/ml), or LPS (100 ng/ml) for 24 hours. Supernatant cytokines were measured and shown as fold mock R848 induction **(B)**. or fold mock LPS induction **(C)**. Cytokines induced by >5-fold mock after R848 stimulation are shown; data bars indicate mean + SEM; asterisks indicate Tukey’s *post hoc* test p<0.05 (two-tailed).

Exposure to TLR ligands can ‘tolerize’ responses to subsequent TLR stimulation in myeloid cells (15–17); other soluble mediators (e.g., cytokines) have also been shown to play a role in systemic immune dysfunction in cancer (18). Alternatively, increased myeloid derived suppressor cells, Tregs, and other immunosuppressive cell types may attenuate monocyte inflammatory responses. To test whether soluble mediators and/or the observed increased TLR ligands in IPMN and PDAC sera (**Figure 3**) are capable of mediating monocyte reprogramming, we incubated healthy donor monocytes with serum from IPMN (n=14), PDAC (n=23), or age-matched healthy donors (n=10) for 48 hours, washed to remove serum constituents, and stimulated with R848 or LPS for 24 hours (**Figure 4A**). **Figures 4B, C** shows cytokines with mean induction >5-fold. Remarkably consistent with *in vitro* whole blood TLR7/8 and TLR4 stimulations using blood from patients with IPMN or PDAC (**Figures 1**, **2**), monocytes previously incubated with sera from patients with IPMN or PDAC induced weaker TNF release compared to that of healthy donors (**Figures 4B, C**). Intriguingly, however, this was selective for TNF, as IL-1β levels were unchanged after IPMN sera exposure but induced after PDAC sera exposure. Other tested cytokines were not different by disease status (IL-6 and GM-CSF), and TNF and GM-CSF induction in IPMN-treated monocytes after LPS stimulation was higher than that of healthy donors (**Figure 4C**). Notably direct analysis of serum content did not reveal significant differences in detected cytokines (IL-1β, IL-6, TNF, CXCL10, IFN-Λ1, IL-8, IFN-α2, IFN-Λ2, IFN-β, or IFN-γ; **Supplementary Figure S7**). Together these data imply functional reprogramming of monocytes observed in IPMN and PDAC is mediated by soluble factors.

## 4 Discussion

In this study, we comprehensively profiled the systemic and local tumor microenvironment of patients with IPMN. We found only a few differences in the tumor immune cell infiltrate between patients with HGD *vs* LGD IPMN. For example, there were greater peripheral blood total T cells and CD4+ T cells in patients with LGD IPMN than HGD IPMN. Unique to our study is the measurement of innate immune cell function. We observed superior TNF responses in monocytes from patients with LGD IPMN after challenge with a TLR7/8 agonist. Similarly, we noted greater TNF responses in patients with stage 1 *vs* stage 2 PDAC. These findings indicate that monocyte reprogramming increases in both higher grade IPMNs and higher stage PDAC.

Since TLR mediated inflammation has been implicated in monocyte reprogramming, we assessed the systemic TLR stimulatory profile in patients using serum samples and TLR reporter cell lines (14). We noted increased TLR3 and TLR4 stimulation by the serum from patients with HGD *vs* LGD IPMN and a trend towards increased TLR stimulation by the serum from stage 2 *vs* stage 1 PDAC. This difference suggests a link between higher TLR-mediated inflammation and decreased monocyte function. Indeed, sera from patients with IPMN and PDAC led to suppressed TNF induction after TLR7/8 stimulation. Future studies are needed to confirm whether this is due to the presence of TLR ligands in sera or other factors. Notably, in both whole blood from patients with IPMN/PDAC as well as IPMN/PDAC patient sera treated healthy donor monocytes, TNF secretion was only impaired after TLR7/8, but not after TLR4, stimulation. This likely indicates that general suppression of the TNF transcription (e.g. epigenetic regulation) or biosynthesis does not explain these differences, but rather may indicate altered TLR-specific cell signaling processes. Indeed, the induction of GM-CSF was higher after TLR4 stimulation in IPMN sera preconditioned monocytes, and levels of IL-1β trended higher in both IPMN and PDAC. Together these data do not necessarily reflect tolerance or desensitization of monocytes to TLR stimuli, but rather reprogramming of the responses elicited by TLR stimulation.

Previous immunophenotyping of IPMNs has focused on describing the local tumor microenvironment. Single cell RNA sequencing efforts by Bernard et al. have suggested that as IPMNs progress from LGD to invasive disease, there is enriched stromal infiltration of myeloid-derived suppressor cells (MDSCs) and these stroma MDSCs acquire a tumor-promoting phenotype (7). Roth et al. utilized immunohistochemical analysis to confirm that macrophages not only increase in the peritumor stroma but also invade regions directly surrounding neoplastic cells as IPMNs progress from LGD to HGD (19). Our findings demonstrate that innate immune rearrangements are systemic, potentially *via* soluble mediators in serum. Monocytes have been previously implicated in cancer propagation and metastasis. In response to the chemoattractant CCL2, monocytes extravasate from the vasculature into primary tumor sites and are reprogrammed within the tumor microenvironment to limit their cytotoxity. These reprogrammed monocytes and monocyte-derived tumor associated macrophages have been shown to promote tumorigenesis by facilitating immune suppression, extracellular matrix remodeling, angiogenesis, and tumor cell migration (14, 20, 21). In patients with PDAC, Sanford et al. described an inverse correlation between monocyte prevalence in the peripheral blood and survival. Additionally, they found that human PDAC tumors had elevated CCL2 mRNA, which initiates monocyte extravasation into tissue, compared to normal pancreas (22). In a mechanistic study, Mitchem et al. targeted monocyte and macrophage tumor infiltration with CSF1R and CCR2 inhibitors in a PDAC mouse model. After CSF1R and CCR2 antagonism, they confirmed a significant reduction in tumor infiltrating monocytes and macrophages and found that tumor size decreased, an enhanced response to chemotherapy, and that hepatic and peritoneal metastasis decreased (23). Our data supports these findings and shows that as IPMN tumors undergo malignant transformation into PDAC, monocytes are also functionally altered.

We previously showed that systemic TLR inflammation can have profound effects on innate immune function, specifically that of monocytes (24). For example, patients with severe COVID-19 demonstrate excessive systemic TLR inflammation which can tolerize monocytes and that these “tolerant” monocytes are unable to respond to subsequent TLR stimulation (14). Similar to the excessive inflammation in COVID, the precancerous and cancer state of IPMN and PDAC may cause excessive TLR-mediated inflammation that reprograms monocytes. Patients with LGD IPMN, which is the least inflammatory stage of pre-cancer, have unaltered monocytes with proficient TNF responses to TLR stimulation. As the stage of the pre-cancerous lesion progresses to HGD IPMN and eventually PDAC, systemic TLR inflammation also increases. We hypothesize that this increase in systemic TLR inflammation results in further monocyte exposure to chronic stimulation, altering their response upon repeat challenge. Concordant with our findings in COVID patients, this study of patients with IPMN and PDAC supports that monocyte reprogramming is associated with poor outcomes.

A significant limitation of our study is the small sample size for the cohort of patients with IPMN. The majority of patients with IPMNs do not proceed to surgical resection so we were unable to study early LGD lesions (without concerning features on imaging). Additionally, the COVID pandemic interrupted the collection and analysis of samples because research activities were temporarily stopped at our institution, leading to a smaller sample size. In general, our institution is high volume in pancreas surgery and only about ten patients with IPMN undergo surgical resection. Thus, our 16 patient samples signify 50% patient accrual during our enrollment time period. Another limitation of our study is the short median follow up on both our IPMN and PDAC cohort. This issue limited our ability to correlate monocyte function and TLR activation with clinical characteristics such as survival. In the future, as this cohort of patients matures, we will adjust our analysis to see if the same trends apply to disease-free and overall survival.

Our observations indicate systemic changes in innate immune function in both HGD IPMN and PDAC. Such changes may have roles in enabling tumor progression and/or serve as prognostic features. Further study into whether peripheral innate immune reprogramming impacts or merely reflects tumor progression are warranted.

## Data availability statement

The raw data supporting the conclusions of this article will be made available by the authors, without undue reservation.

## Ethics statement

The studies involving human participants were reviewed and approved by Duke Health IRB. The patients/participants provided their written informed consent to participate in this study.

## Author contributions

AE, MB, KL, IN, EH, DB, PA and SN contributed to conception and design of the study. SN administered the project and reviewed data. AE, MB, KL, IN, BS, GB and SN interpreted data. AE, AF and KL consented patients and biobanked surgical specimens. AE, MB, KL, IN, DB, MG, AF and KR carried out the experiments. AE, MB and IN performed the statistical analysis. AE, MB, SN and IN contributed towards manuscript writing. All authors contributed to manuscript revision and read and approved the submitted version.
