# Supplementary material for: Functional reprogramming of peripheral blood monocytes by soluble mediators in patients with pancreatic cancer and intraductal papillary mucinous neoplasms
Source: Front Immunol. 2023 Jul 28;14:1116034. doi: 10.3389/fimmu.2023.1116034 (PMC10416516; doi:10.3389/fimmu.2023.1116034)
Supplement: Supplementary file 1 [file DataSheet_1.pdf]

Supplementary Material

Functional Reprogramming of Peripheral Blood Monocytes by Soluble Mediators in Patients with Pancreatic Cancer and Intraductal Papillary Mucinous Neoplasms

Austin Eckhoff MD[1†], Michael C. Brown PhD[2†], Karenia Landa MD[1], Ibtehaj Naqvi MD PhD[3], Eda K. Holl PhD[1], Ashley Fletcher BS[1], David Boczkowski MS[1], Kristen E. Rhodin MD[1], Minh Huy Giang BS[4], Bruce Sullenger PhD[1], Georgia M. Beasley MD[1], Peter J. Allen MD[1], Smita K. Nair PhD[1,2,4*]

1 Department of Surgery, Duke University; Durham, North Carolina, USA
2 Department of Neurosurgery, Duke University; Durham, North Carolina, USA
3 Department of Anesthesiology, Duke University; Durham, North Carolina, USA
4 Department of Pathology, Duke University; Durham, North Carolina, USA

† Equal contribution and first authorship

* Correspondence:
Smita K. Nair
smita.nair@duke.edu

Supplementary Figure 1 (Figure S1)

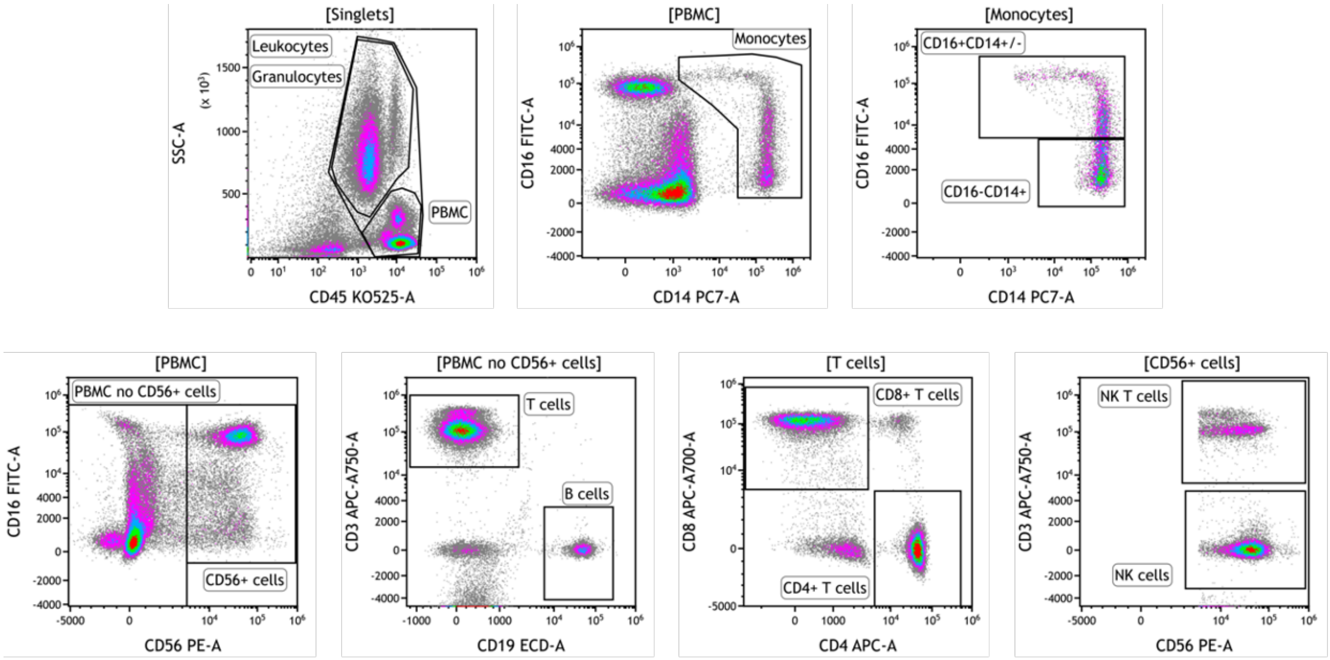

Figure S1. Flow cytometry gating strategy used to analyze immune cell subsets in blood.
Figure shows flow cytometry gating strategy for manuscript Figures 1 and 2. Whole blood was obtained from cancer patients and analyzed within 2 hours of collection The gates are indicated on top of each population and cell types are identified within each panel. Leukocytes in singlets were gated based on expression of CD45. Leukocytes were further separated as granulocytes (large cells based on side scatter) and peripheral blood mononuclear cells (PBMC), which includes lymphocytes and monocytes. Cells were separated based on expression of surface markers (CD14, CD16, CD19, CD3, CD56, CD4, and CD8). Monocyte subsets were identified based on CD14 and CD16 cell surface expression; CD16–CD14+ classical monocytes and CD16+CD14+/– intermediate and non-classical monocytes. PBMC were separated into PBMC with no CD56+ cells and CD56+ cells (which includes NK and NK T cells). PBMCs with no CD56+ cells (PBMC no CD56+ cells) were then separated into CD3+ T cells and CD19+ B cells. CD3+ T cells were then separated into CD8+ T cells and CD4+ T cells. CD56+ cells were further separated into CD56+CD3– NK cells and CD56+CD3+ NK T cells.

Supplementary Figure 2 (Figure S2)

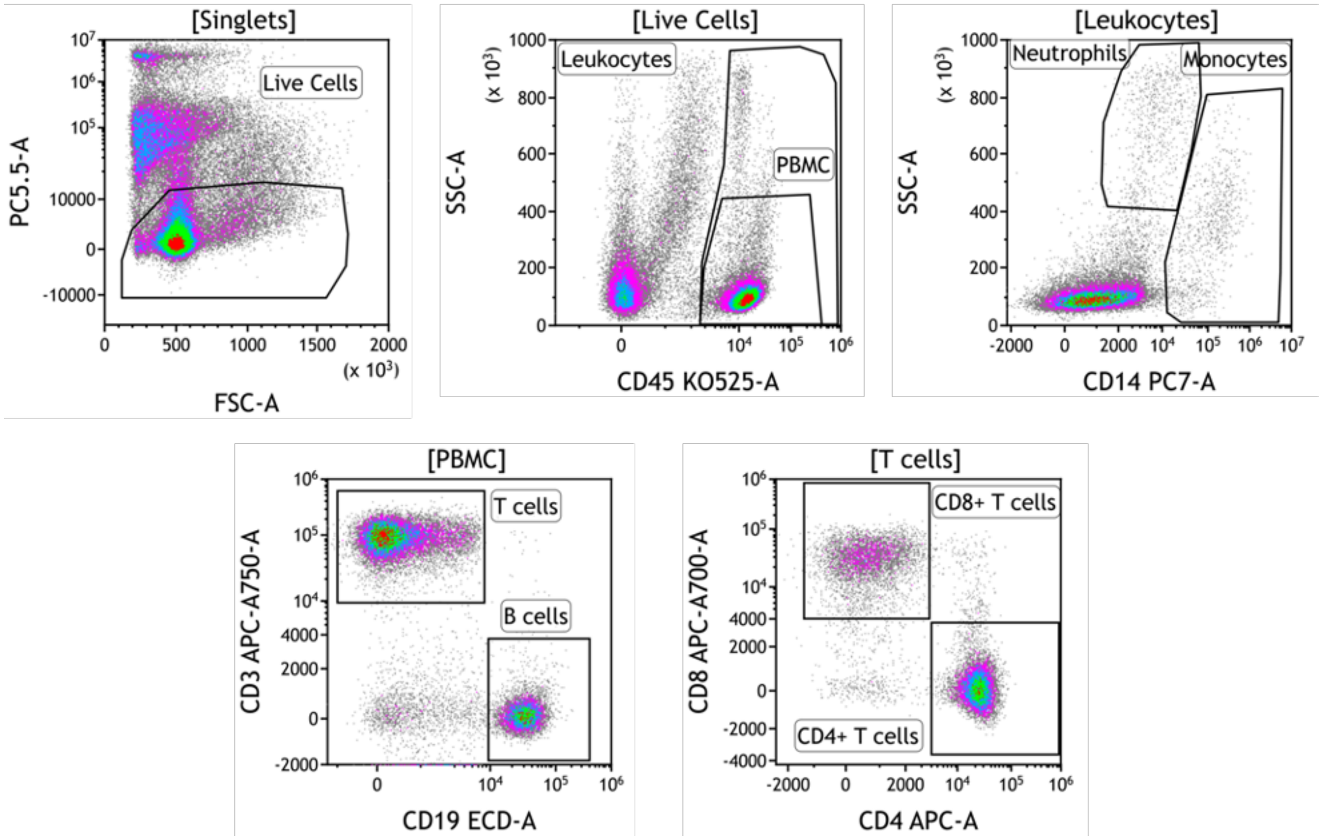

Figure S2. Flow cytometry gating strategy used to analyze immune cell subsets in tumor tissue. Figure shows flow cytometry gating strategy for manuscript Figures 1 and 2. The gates are indicated on top of each population and cell types are identified within each panel. Tumor tissue was digested and gated on live cells (propidium iodide (PI)-negative). Leukocytes and PBMC within leukocytes were identified based on expression of CD45. Immune cell subsets were separated based on expression of surface markers (CD14, CD16, CD19, CD3, CD4, and CD8). T and B cells in PBMC were identified based on expression of CD3 (T cells) and CD19 (B cells). CD3+ T cells were separated into CD4+ T cells and CD8+ T cells.

Supplementary Figure 3 (Figure S3)

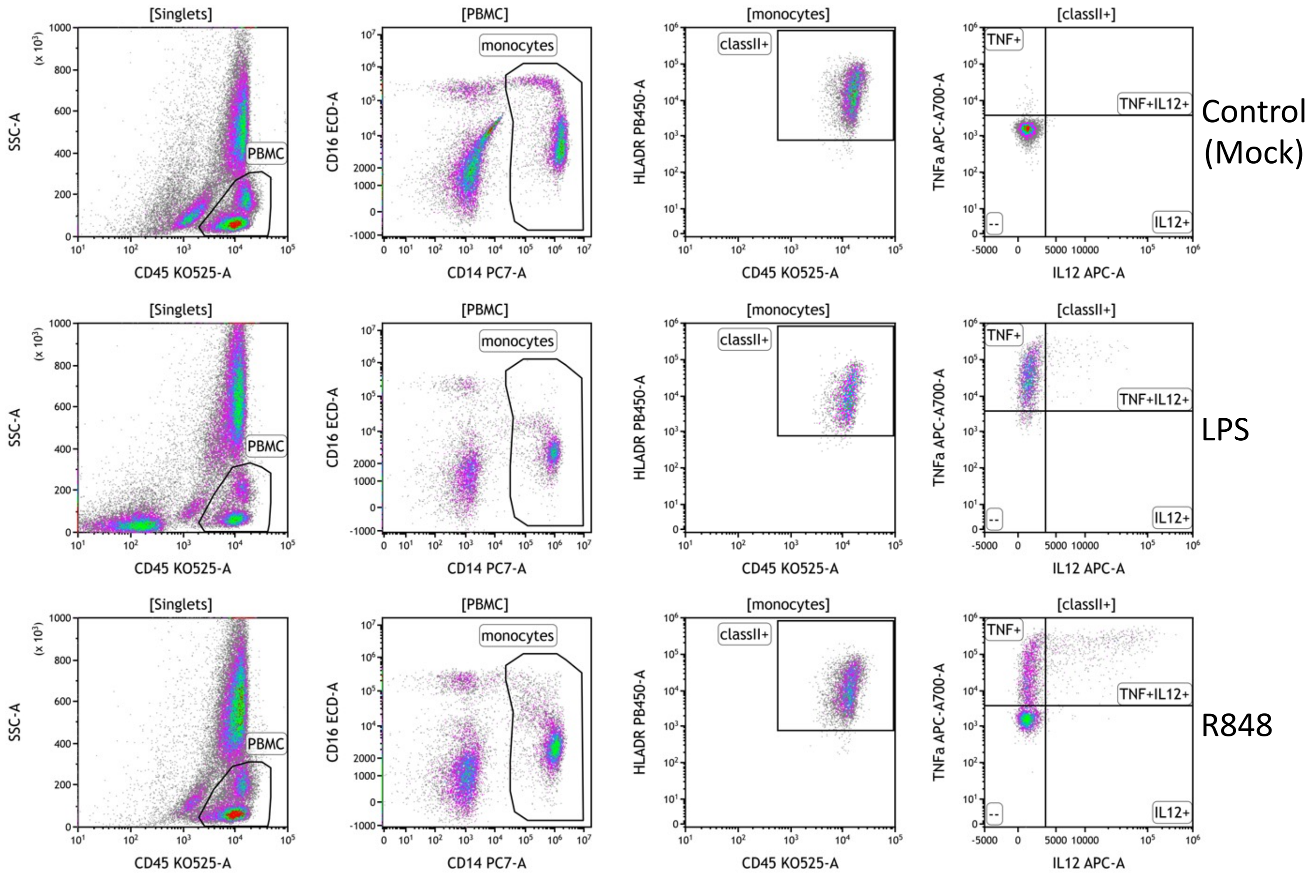

Figure S3. Analysis of monocyte function in whole blood.

Whole blood was either untreated (mock) or treated with the pattern recognition receptor (PRR) agonists, LPS (lipopolysaccharide; TLR4 agonist) or R878 (TLR7/8 agonist) for 6 hours. Figure shows flow cytometry gating strategy for manuscript Figures 1 and 2. The gates are indicated on top of each population and cell types are identified within each panel. PBMC were identified within CD45+ leukocytes. Monocytes were identified based on expression of CD14 and CD16 and MHC class II expression on monocytes was confirmed. TNF and IL12 production in class II+ monocytes was measured using intracellular cytokine staining.

Supplementary Figure 4 (Figure S4)

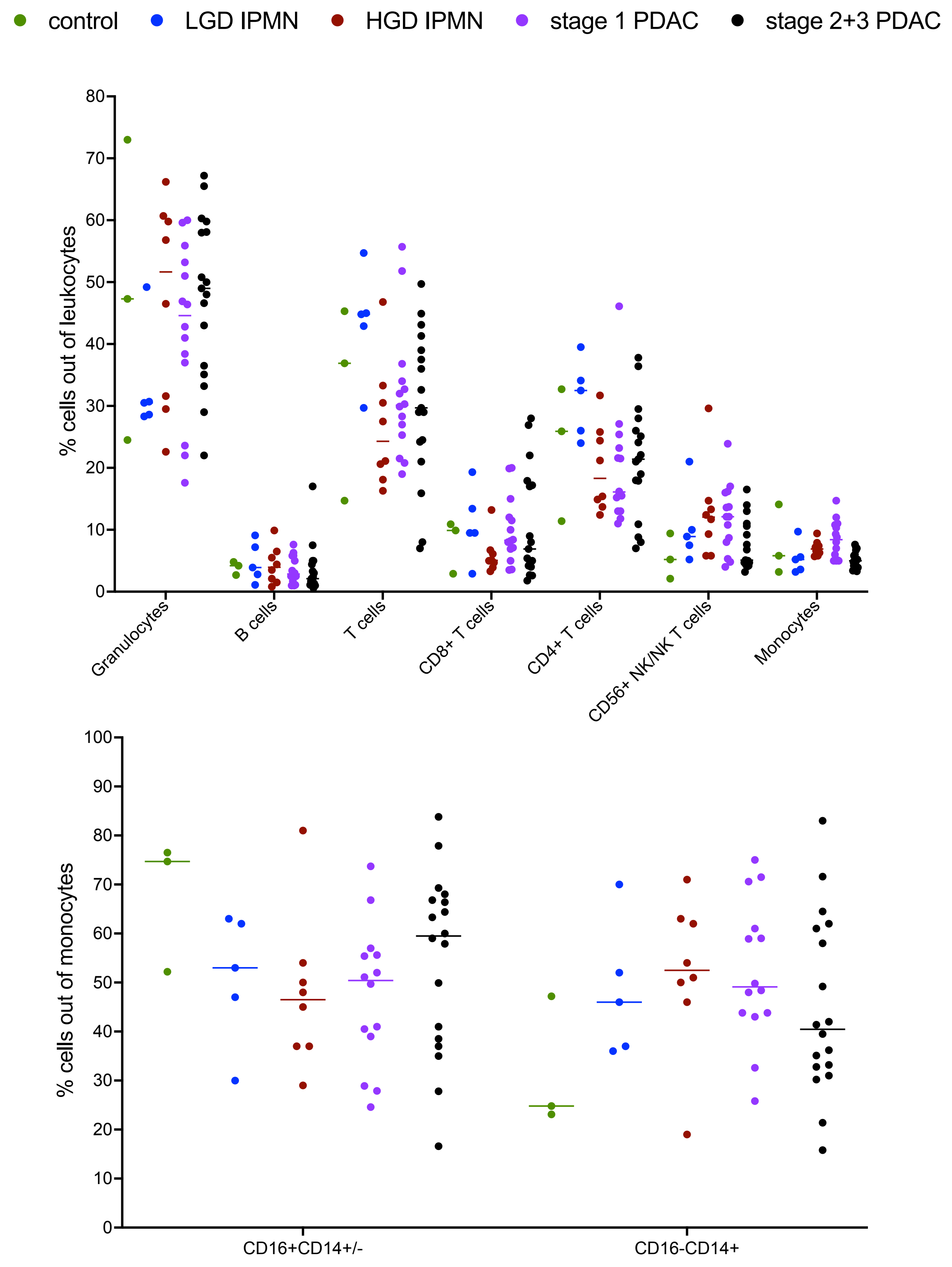

Figure S4. Analysis of immune cell subsets in peripheral blood in patients with IPMN and PDAC.
Top panel: Flow cytometry analysis of immune cells subsets in blood from healthy volunteer (control), LGD IPMN, HGD IPMN, stage 1 PDAC and stage 2+3 PDAC.
Bottom panel: Comparison of monocyte subsets (CD16+CD14+/- and CD16-CD14+) in blood from healthy volunteer (control), LGD IPMN, HGD IPMN, stage 1 PDAC and stage 2+3 PDAC.

Supplementary Figure 5 (Figure S5)

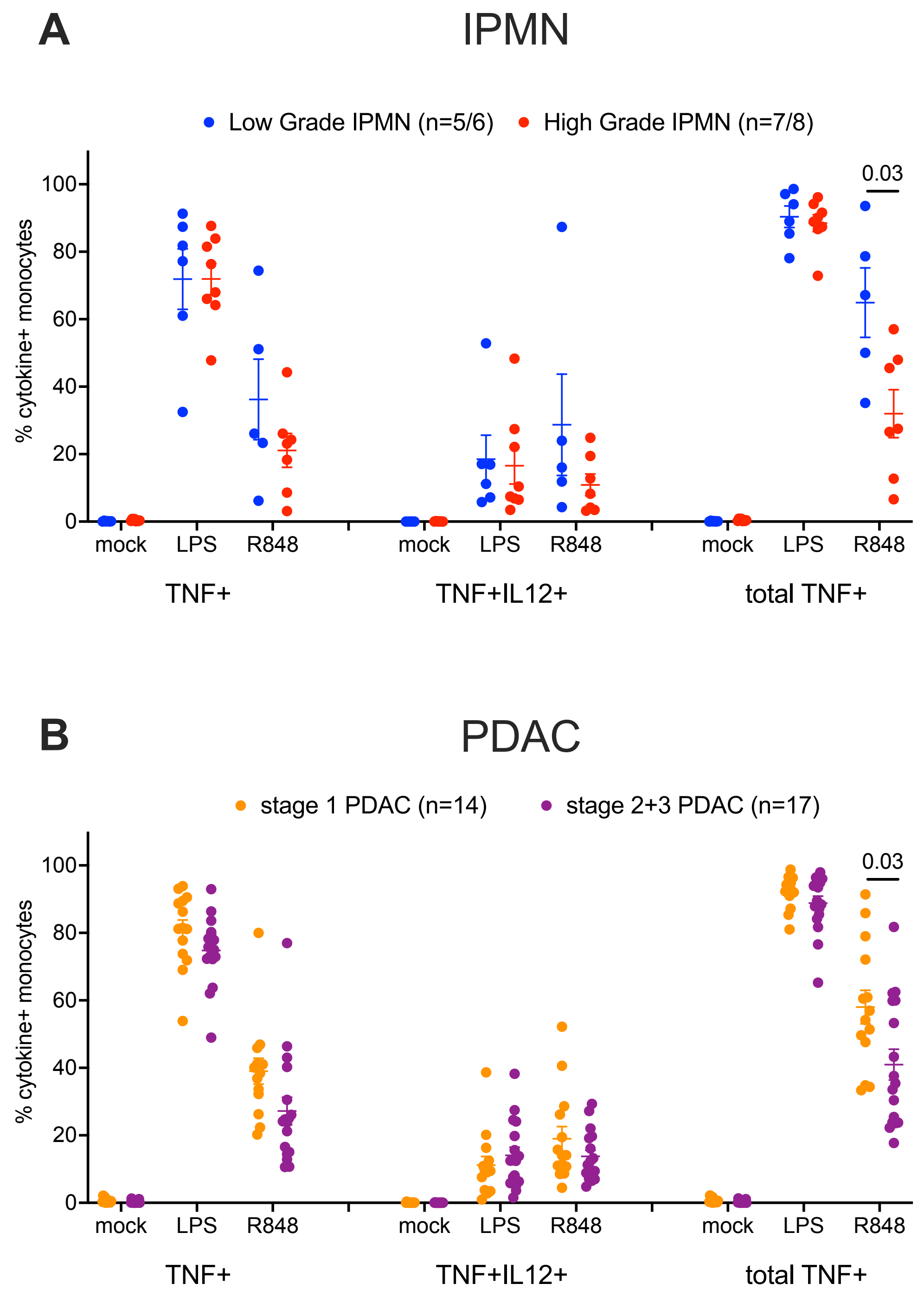

Figure S5. Analysis of monocyte function in whole blood.
Extended flow cytometry analysis data for Figures 1D and 2D. Whole blood was either untreated (mock) or treated with the pattern recognition receptor (PRR) agonists, LPS (lipopolysaccharide; TLR4 agonist) or R878 (TLR7/8 agonist) for 6 hours. Figure S3 shows flow cytometry gating strategy for manuscript Figures 1 and 2. TNF and IL12 production in class II+ monocytes was measured using intracellular cytokine staining. Although we did find TNF and IL12 producing cells, we did not observe any cells that produced IL12 alone. We have therefore not shown IL12 producing cells in Figure S5. Figure shows individual values with mean +/- SEM.

Supplementary Figure 6 (Figure S6)

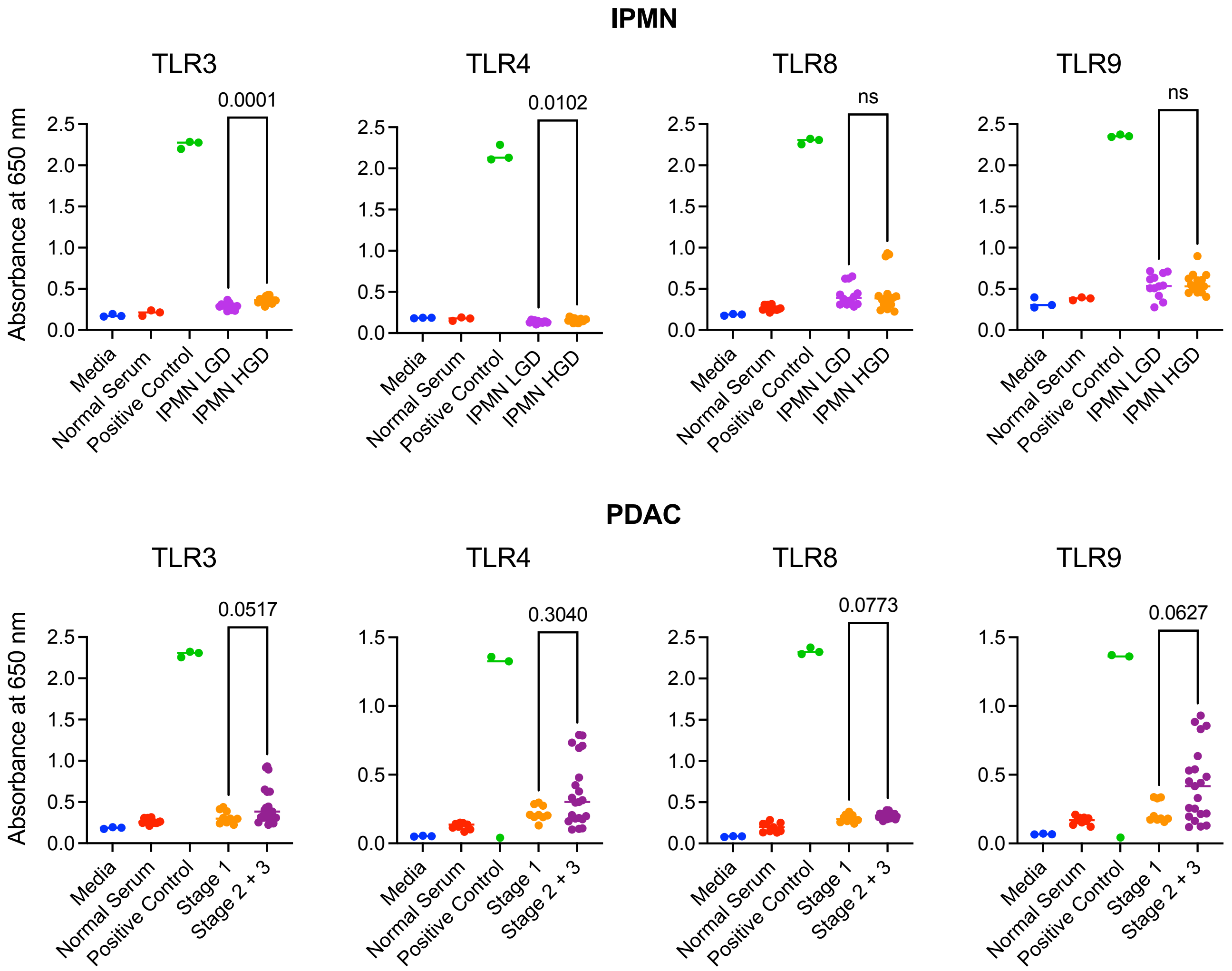

Figure S6. TLR activation assay.

Extended data from Figure 3 presented with experimental negative (media alone) and positive (TLR3 = Poly I:C, TLR4 = LPS, TLR7 = R848, and TLR9 = CpG) controls. In all cell lines, positive controls induced more than adequate stimulation of the reporter cells while media alone induced minimal stimulation. Comparison of the ability of serum from patients with LGD vs HGD and patients with stage 1 PDAC vs stage 2/3 PDAC to stimulate TLRs 3, 4, 7, and 9. P-values shown obtained with Mann-Whitney test.

Supplementary Figure 7 (Figure S7)

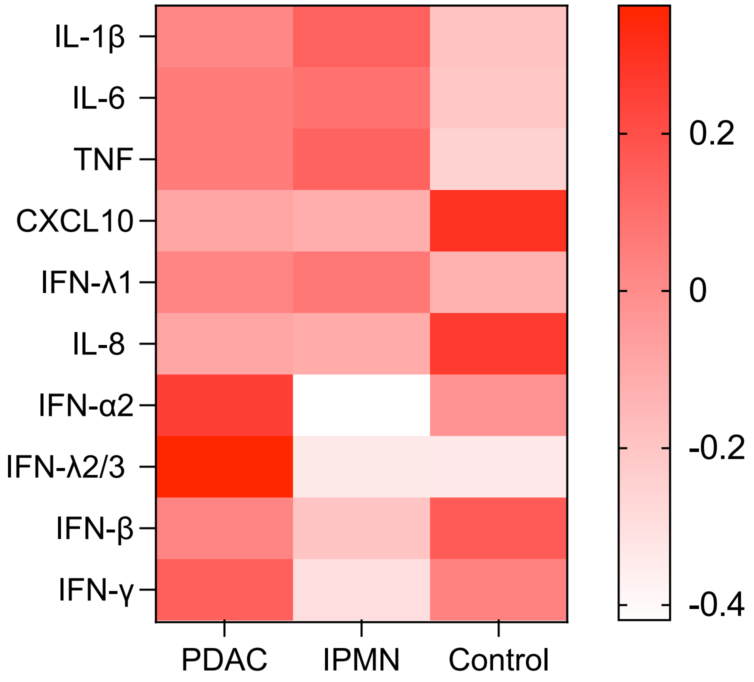

Figure S7. Cytokine analysis of serum from patients with IPMN, PDAC, versus age matched healthy donors. Median florescence intensity values for each analyte were converted to z-scores; sera samples correspond to the sera used to precondition healthy donor monocytes in Figure 4.
